# Implementation of minimally invasive gastrectomy for gastric cancer in a western tertiary referral center

**DOI:** 10.1186/s12893-020-00812-w

**Published:** 2020-07-16

**Authors:** Andrianos Tsekrekos, Tania Triantafyllou, Fredrik Klevebro, Masaru Hayami, Mats Lindblad, Magnus Nilsson, Lars Lundell, Ioannis Rouvelas

**Affiliations:** 1 Department of Upper Abdominal Surgery, Karolinska University Hospital, Stockholm, Sweden; 2 Division of Surgery, Department of Clinical Science, Intervention and Technology (CLINTEC), Karolinska Institutet, Stockholm, Sweden; 3 1st Propaedeutic Surgical Clinic, Hippocration General Hospital, Athens, Greece; 4 Department of Surgery, Odense University Hospital, Odense, Denmark

**Keywords:** Gastric cancer, Minimally invasive surgery, Laparoscopic gastrectomy

## Abstract

**Background:**

Minimally invasive techniques have gradually come to take a leading position in the surgical treatment of gastrointestinal malignancies. In order to define an effective process for the implementation of similar techniques in the treatment of gastric cancer, patient caseload represents a pivotal factor for education and training, but is a prerequisite not fulfilled in most Western countries. Additionally, as opposed to the East, a variety of additional factors such as the usually advanced stage of the disease and differences in patient characteristics are prevailing and raise further obstacles. Hereby we report a strategy for a safe and effective process for the implementation of laparoscopic gastric cancer surgery in a Western tertiary referral center.

**Methods:**

The present study describes the stepwise implementation of laparoscopic gastrectomy for the treatment of gastric cancer at a tertiary referral center, comprising the time period 2012–2019. This process was facilitated by a close collaboration with two high-volume centers in Japan, as well as exchanging fellowships and observerships between the Karolinska University Hospital and other European centers. From the initially strict selection of cases for laparoscopic surgery, laparoscopic gastrectomy has gradually become the preferred approach also in patients with locally advanced tumors.

**Results:**

From January 1st 2010 until December 31st 2019, 249 patients were operated for gastric cancer, of whom 141 (56.6%) had an open and 108 (43.4%) a laparoscopic procedure. In the latter group, total gastrectomy was performed in 33.3% of the patients. While blood loss, operation time and length of stay decreased during the first years after implementation, these variables increased slightly during the last years of the study period, probably due to the higher proportion of advanced gastric cancer cases, as well as the higher rate of laparoscopic total gastrectomy with more extended lymphadenectomy.

**Conclusions:**

Laparoscopic surgery is currently a valid therapeutic option for gastric cancer, which has expanded to also embrace total gastrectomy and locally advanced tumors. Collaboration between centers in the East and West, centralization to high-volume centers and application of enhanced recovery protocols are essential components in the implementation and further refinement of minimally invasive gastrectomy.

## Background

Gastric cancer remains the third leading cause of cancer-related mortality worldwide [1]. During the last decades, high incidence East Asian countries have established targeted screening programs for early detection of gastric cancer and intensive endoscopic surveillance of suspicious lesions. Subsequently, in parity with more frequent diagnosis at an earlier stage, minimally invasive therapeutic techniques have also evolved and are now widely applied, including endoscopic resection of early tumors carrying a very low risk for lymph node metastases and laparoscopic surgery for stage I disease [2, 3]. In Western countries, only a small fraction of patients fulfill those criteria at diagnosis. The majority still present at an already advanced stage of the disease, where surgical resection with adequate D2 lymphadenectomy, usually in combination with perioperative chemotherapy, remains the gold standard of treatment [4]. However, despite several advances, 5-year survival on a population-based level is only achieved in approximately 30% of patients [5].

Aiming to improve the management of patients with gastric cancer, a multidisciplinary approach comprising a number of different strategies has been implemented in many institutions. Identification and elimination of morbidity related risk factors such as alcohol consumption and tobacco smoking, significant advances in anesthesiology, constant nutritional support, enhanced recovery after surgery and fast-track protocols have positively affected the surgical outcome. Another element of this process is the implementation of minimally invasive surgical techniques, such as laparoscopic and robotic-assisted gastrectomy, which aim to minimize the surgical trauma and accelerate the postoperative recovery. Several studies have demonstrated the advantages of laparoscopic surgery in early gastric cancer (EGC) and there are indications that this may also apply in advanced gastric cancer (AGC) [6, 7]. Nevertheless, locally advanced tumors require a more extensive lymph node dissection, representing a technically more demanding procedure. As the gastric cancer surgical caseload is significantly higher in the East, with superior results constantly reported, extrapolation of the Eastern technical expertise to the West may be the key for further improvement of the outcomes.

The aim of this study is to present our unit’s experience on implementing minimally invasive gastrectomy for the treatment of gastric cancer, which occurred in collaboration with two high-volume centers in Japan.

## Methods

The current study is a description of the process of stepwise implementation of the laparoscopic technique for the surgical treatment of gastric cancer at a tertiary referral center. All patients diagnosed with gastric cancer and treated with curative intent at the Karolinska University Hospital over the period 2010–2019 were identified in the hospital’s surgical planning system (ORBIT) and cross-matched for validation with the patient chart system (Take Care). A small number of patients (*n* = 7) subjected to pylorus preserving gastrectomy were excluded. Data regarding clinical tumor stage (cTNM), type of gastrectomy (open or laparoscopic, distal or total), intraoperative blood loss, operation time, number of retrieved lymph nodes and length of hospital stay were extracted by reviewing the patients’ charts.

### Description of the implementation process over time

#### Initial phase

Table 1 summarizes the different steps of implementation of laparoscopic gastrectomy for gastric cancer at the Karolinska University Hospital, which basically occurred in parallel with the implementation of minimally invasive esophagectomy by the same team of surgeons, and which has also been reported recently [8]. In August 2012, our department established a 2-year Upper Gastrointestinal Surgery fellowship program with visiting Japanese gastric surgeons in collaboration with the Cancer Institute Hospital and Keio University in Tokyo, Japan. One of the goals of this collaboration was the incorporation of the surgical expertise of the Japanese fellows in our surgical practice.

**Table 1:** Steps in the introduction of laparoscopic gastrectomy for gastric cancer at Karolinska University Hospital

Step	Time period	Implementation of laparoscopic gastrectomy
1	2009–2012	Consultant surgeon IR, 3-year fellowship in minimally invasive UGI-surgery, Bristol Royal Infirmary, Bristol, UK
2	February 2011	Consultant surgeon MN visits Cancer Institute Hospital, Tokyo, Japan
3	March 2012	Consultant surgeons MN & ML visit center with experience in the field of minimally invasive UGI-surgery, AMC, Amsterdam
4	August 2012	Establishment of recurring 2-year fellowship (Japanese gastric surgeon) at Karolinska, in collaboration with the Cancer Institute Hospital, Tokyo, Japan (ongoing)
5	September 2012	First case of laparoscopic distal gastrectomy
6	2012–2013	Consultant surgeon ML, 1-year surgical training in minimally invasive UGI-surgery at Flinders Medical Center, Adelaide, Australia
7	April 2013	First case of laparoscopic total gastrectomy
8	May 2015	First case of laparoscopic total gastrectomy with functional end-to-end anastomosis using linear stapler

*UGI-surgery* upper gastrointestinal surgery, *AMC* Academic Medical Center

The first laparoscopic distal gastrectomy (LDG) was performed in September 2012. During the initial phase of the implementation period, only distal gastrectomies were performed with the laparoscopic technique in selected patients with clinical stage I disease (i.e. T1N0, T1N1 or T2N0) in accordance with the Japanese gastric cancer treatment guidelines, excluding patients with large tumors or bulky lymph node metastases. This selection was due to initial concerns regarding the completeness of lymph node dissection in locally advanced cases and anticipated technical difficulties in performing the esophagojejunal anastomosis. During that period of fine-tuning the technique, more advanced cases were still assigned to conventional open gastrectomy.

#### Extension of indications and evolution of the surgical technique over time

In December 2012, a systematic review and meta-analysis on the short-term outcomes of laparoscopic total gastrectomy (LTG) versus open total gastrectomy (OTG) for gastric cancer indicated that LTG was associated with a significant reduction of intraoperative blood loss, reduced risk of postoperative complications and shorter hospital stay, albeit at the cost of a longer operation time. In-hospital mortality rates were comparable for LTG and OTG [9]. Interestingly, five out of eight studies in this meta-analysis included also patients with AGC, with two of the studies originating from Europe. These two studies, although small, were also the ones with a prospective design [10, 11]. After having performed a sufficient number of LDG without experiencing any technical problems our indications were further extended to also include patients in need of a total gastrectomy, with the first LTG performed in April 2013.

The first three cases of LTG (2013–2014) were selected so that no D2 lymph node dissection was needed (two patients had proximal GIST and one patient underwent prophylactic resection due to *CDH-1* mutation). In these first three LTG cases we used a circular stapling device for the esophagojejunal anastomosis, but soon switched to a simplified technique, using a cutting linear stapler instead to construct a stapled side-to-side anastomosis. This technique was to some extent similar to the one evolved and refined for the intrathoracic anastomosis during the transition period to minimally invasive Ivor Lewis procedure for esophageal cancer in our department and has been described in detail previously [12]. Having demonstrated that this technique is safe and with good results, we could easily apply it also to LTG, with the first operation for gastric cancer performed in May 2015.

Consequently from 2015 and onwards, with the experience acquired by the LDG and having mastered the laparoscopic technique for esophagojejunostomy, virtually all patients were considered candidates for the laparoscopic approach, including those with proximal gastric cancer or Siewert III junctional cancer. However, in diffuse type carcinomas and in cases where the intraoperative findings revealed tumor growth extending beyond the serosa (cT4a), surgery was converted to open, in order to perform bursectomy in accordance with clinical practice at that time. The same applied for locally advanced tumors (cT4b), requiring a multivisceral resection. However, from May 2017 and onwards and based on Japanese data indicating no survival benefit of bursectomy [13], all patients eventually qualified for laparoscopic resection regardless disease stage as long as an R0 result could be achieved. This applied even for patients who required multivisceral resection such as splenectomy with or without distal pancreatectomy.

## Results

Between January 1st 2010 and December 31st 2019, 256 patients underwent surgery for gastric cancer at our department. Seven patients that had a pylorus preserving gastrectomy for EGC were excluded from further analysis. Of the remaining 249 patients, 141 (56.6%) underwent open and 108 (43.4%) laparoscopic gastrectomy. The majority of the patients in the open group had a total gastrectomy (58.9%), compared to 33.3% in the laparoscopic group.

**Fig. 1:**
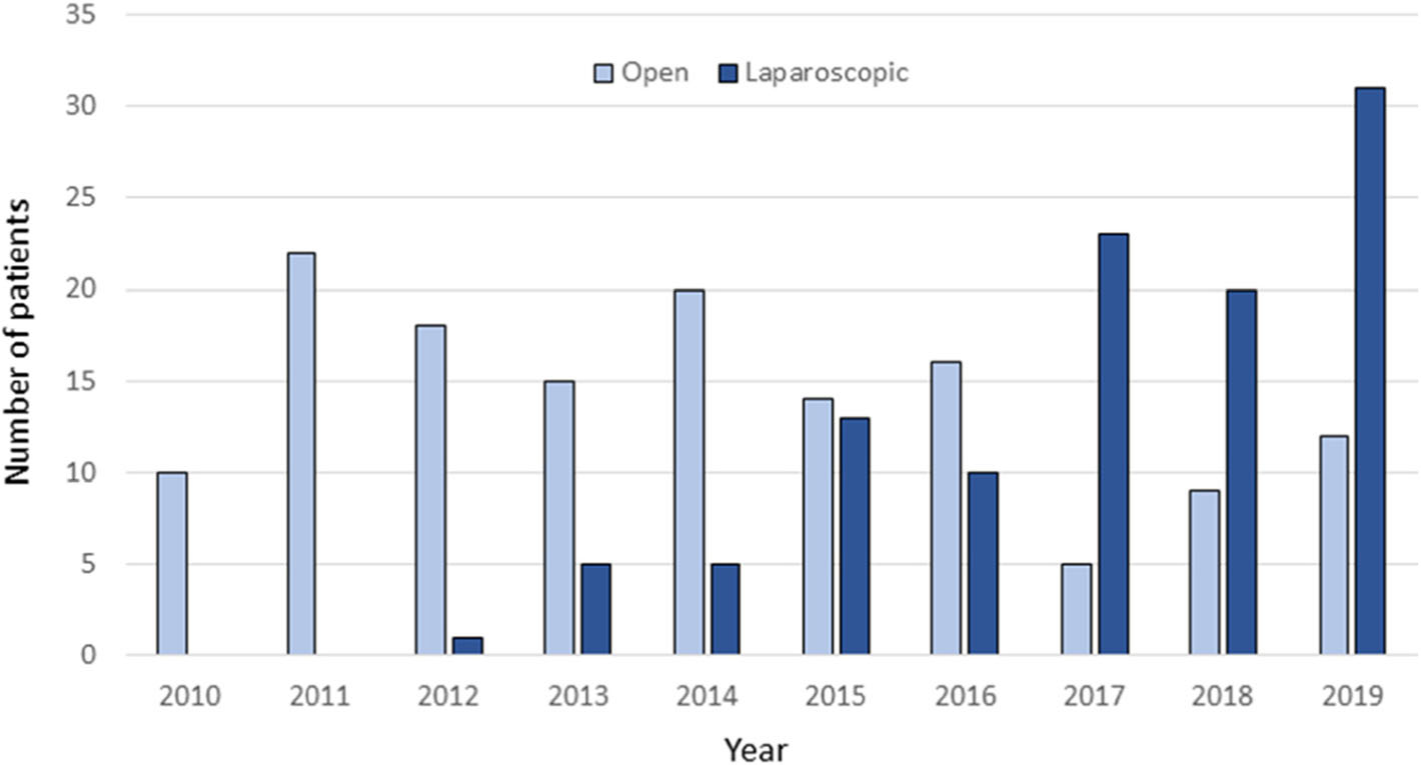
The annual number of open and laparoscopic gastrectomies during the time period 2010–2019 (all cases)

**Fig. 2:**
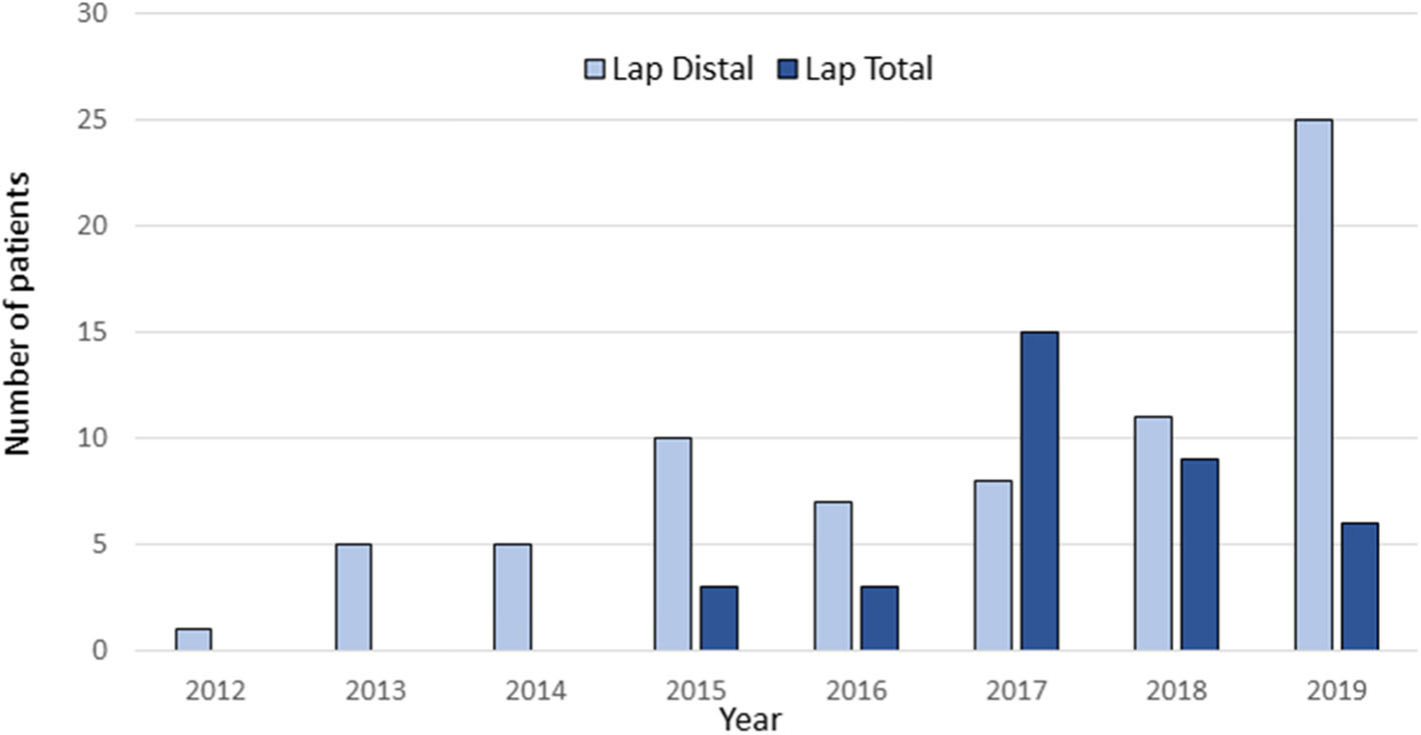
Trend of laparoscopic surgical procedures over time (only cancer cases)

**Fig. 3:**
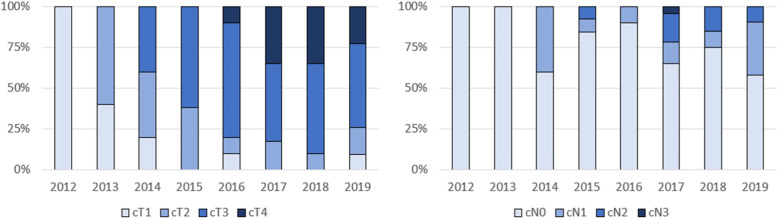
Proportion of patients’ clinical tumor stage (laparoscopic cases only)

During the study period there was a gradual increase in the proportion of laparoscopic procedures and from 2017 and onwards, approximately 75% of all gastrectomies were performed laparoscopically (Fig. 1). In 2015, total gastrectomies also started being performed laparoscopically with an increasing utilization from 2017 (Fig. 2). Over time, there was a gradual increase in the proportion of patients with more advanced tumors (cT2–4 and cN+) that were offered a laparoscopic operation (Fig. 3).

**Fig. 4:**
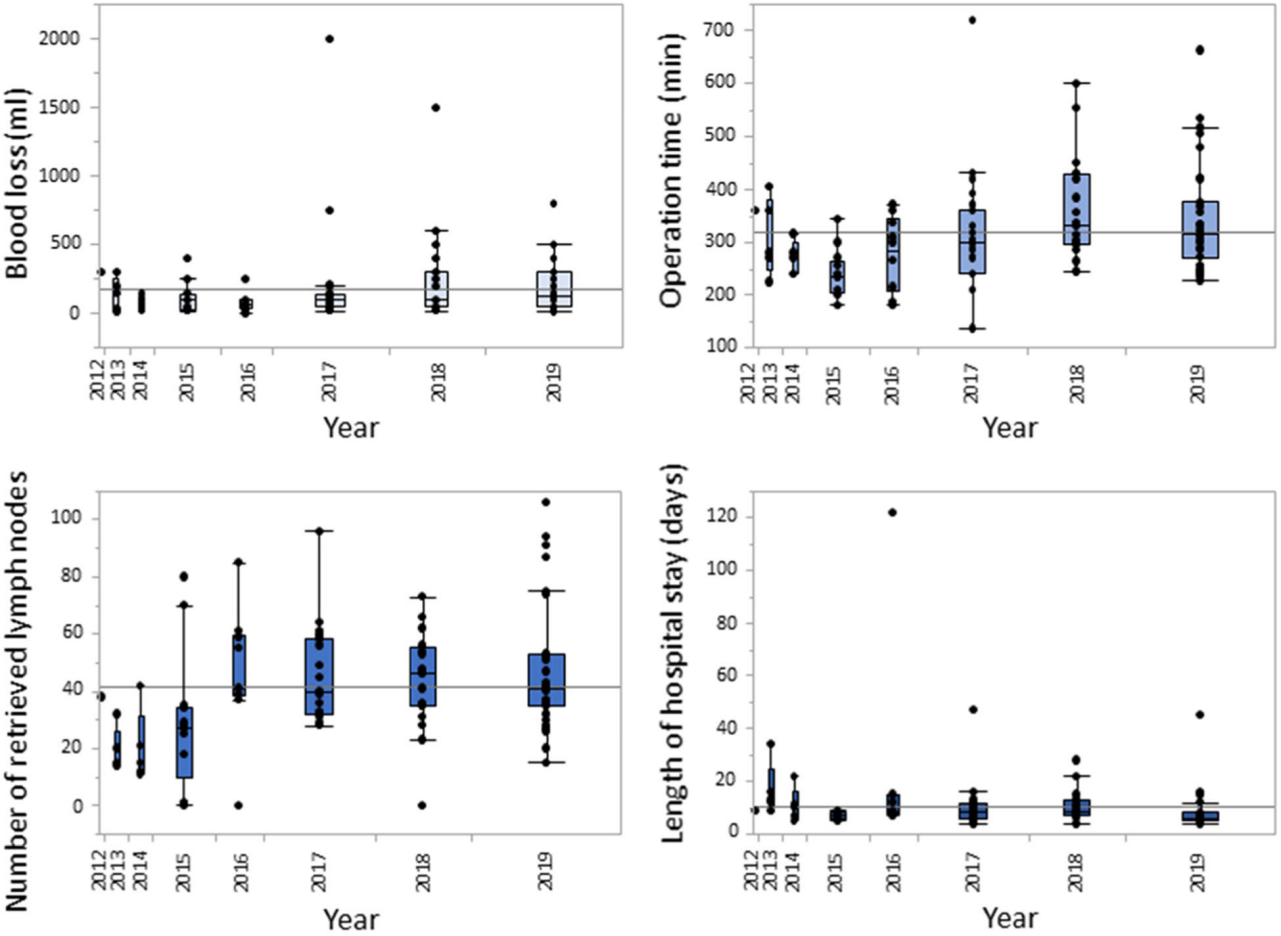
Whisker boxplots illustrating the intraoperative blood loss, operation time, number of retrieved lymph nodes and length of hospital stay over time (laparoscopic cases only)

**Fig. 5:**
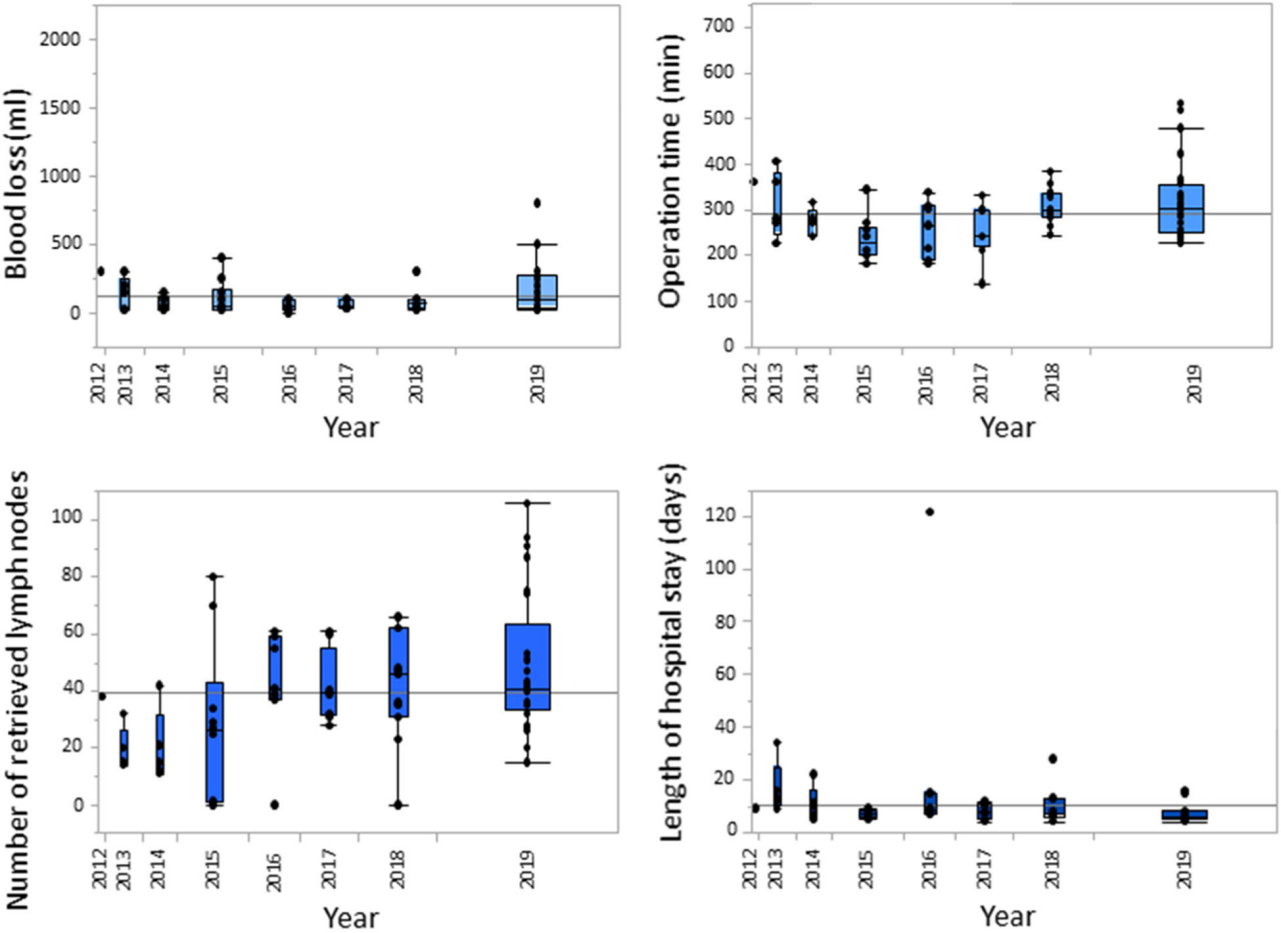
Whisker boxplots illustrating the intraoperative blood loss, operation time, number of retrieved lymph nodes and length of hospital stay over time (laparoscopic distal gastrectomies only)

**Fig. 6:**
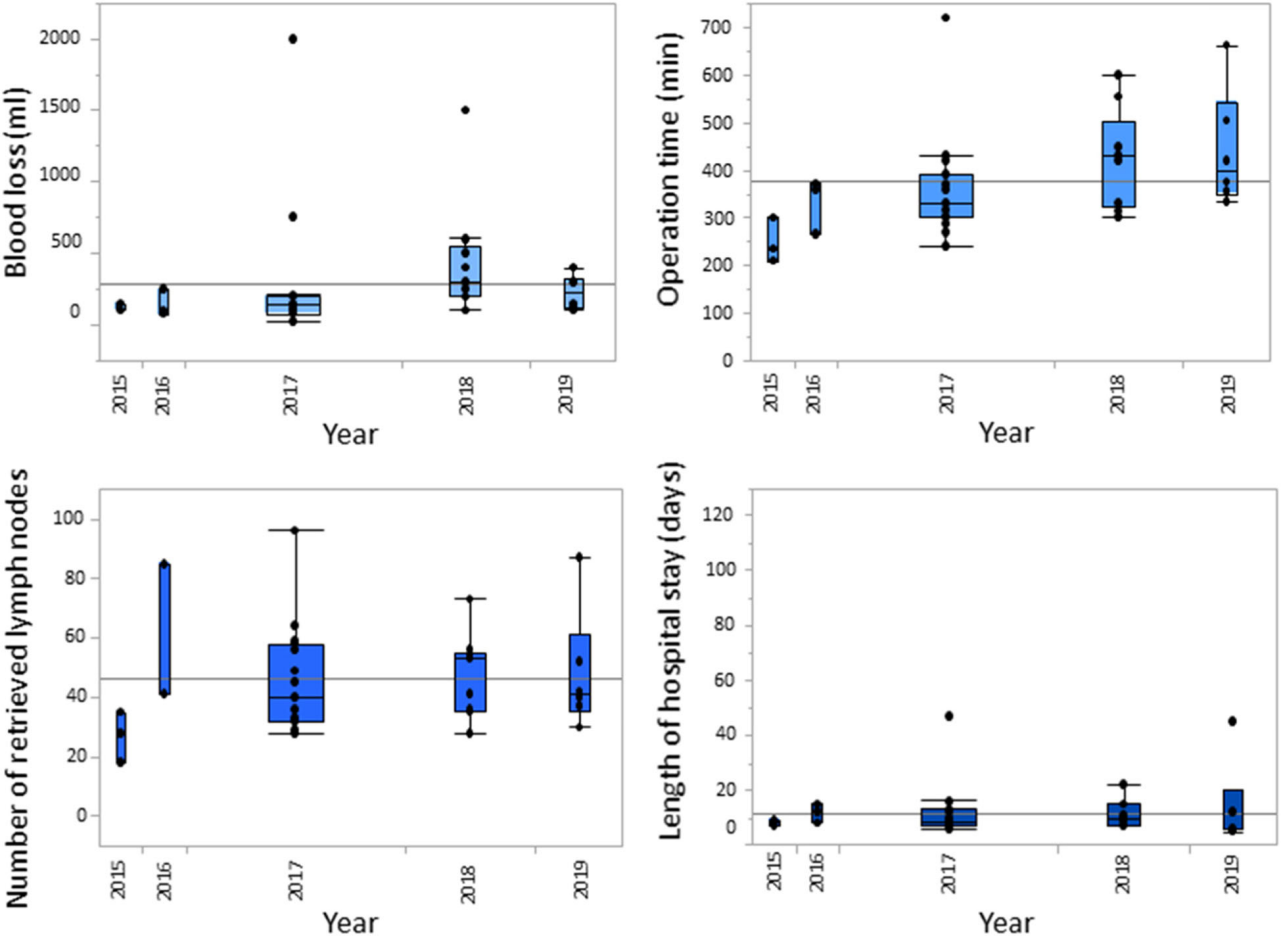
Whisker boxplots illustrating the intraoperative blood loss, operation time, number of retrieved lymph nodes and length of hospital stay over time (laparoscopic total gastrectomies only)

Figure 4 summarizes the data on intraoperative blood loss, operation time, number of retrieved lymph nodes and length of hospital stay for the laparoscopic group. While blood loss, operation time and length of stay decreased during the first years after implementation of LG, these variables leveled off and ultimately increased slightly during the last years, probably related to the increasing proportion of LTGs and AGCs. The same trend in the above-mentioned variables was also observed when the outcomes after LDG and LTG were analyzed separately (Figs. 5 and 6).

## Discussion

Since the early 1990s, when laparoscopic surgery for gastric cancer was first reported, minimally invasive procedures such as endoscopic, laparoscopic and robotic have been gradually introduced in clinical practice and are continuously tested in clinical trials.

LDG is now a valid option for early stage tumors located in the middle or lower third of the stomach; a large number of studies have confirmed the safety of the procedure and shown better short-term outcomes compared to open surgery. Two randomized controlled trials comparing laparoscopic and open distal gastrectomy for stage I gastric cancer have recently reported on their long-term results; KLASS-01 from Korea showed no difference in 5-year overall survival, and JCOG0912 from Japan found no difference in 5-year relapse free survival between the two groups. By demonstrating similar oncologic outcomes, these studies have confirmed the noninferiority of laparoscopic surgery, supporting its adoption as standard treatment [14, 15].

As expected, these results have led to an expansion of the indications of LDG and evidence is emerging on the comparable results of this approach even in AGC. The CLASS-01 trial is the first RCT to show similar 3-year disease-free survival (DFS) between laparoscopic and open distal gastrectomy for patients with AGC [16]. The KLASS-02 has a similar design with 3-year DFS as the primary endpoint and the short-term outcomes have been reported, showing several benefits of the laparoscopic approach, including fewer complications, faster recovery and shorter hospital stay [17]. Additionally, the mean number of retrieved lymph nodes did not differ between the two approaches. Finally, the phase II part of the JLSSG0901 trial (with the first 180 enrolled patients) has demonstrated the safety of LDG with D2 lymph node dissection, with low occurrence of anastomotic leakage and pancreatic fistula [18]; the phase III extension of the trial to confirm the oncological non-inferiority of the procedure is ongoing.

When it comes to LTG, the scientific evidence is scarce; a Japanese retrospective nationwide study by Sakamoto et al., including patients with clinical stage I-III gastric cancer, confirmed the advantages of LTG in terms of time to first oral intake and length of hospital stay; however, the study highlighted a higher incidence of anastomotic leakage after LTG when compared to OTG. Despite this finding, no difference in in-hospital mortality was found [19]. The higher incidence of anastomotic leakage is not surprising, as laparoscopic construction of the esophagojejunostomy is technically more challenging, which has also been demonstrated in a population-based cohort study from the Netherlands [20]. Reporting of five-year oncological outcomes after LTG is even more limited. Nevertheless, a recent large-scale meta-analysis by Oh et al. including 19 studies – with subgroup analysis of EGC and AGC – has shown comparable 5-year survival rates [6]. A number of RCTs comparing LTG with OTG are planned or ongoing and will hopefully provide evidence of higher grade [21, 22].

Although the evidence on laparoscopic gastric cancer surgery is mostly generated in Asia, a number of European studies have also been conducted in recent years. This is important, as European and Asian populations differ in their characteristics; in the West, the disease is often diagnosed at a more advanced stage, with a higher proportion of proximal tumors or tumors of poorly cohesive histologic type. Additionally, patients are older and with a higher Body Mass Index in average, making surgery more challenging [23, 24]. A recent review by Chevallay et al., summarizing the results of 14 studies published from European centers between 2005 and 2017, confirmed the superiority of the laparoscopic approach in terms of less intraoperative blood loss and shorter hospital stay, with no significant difference in the number of the lymph nodes resected, rate of anastomotic leakage or mortality [25]. Nevertheless, laparoscopic gastrectomy still remains more time-consuming, a finding that is consistently reported in both European and Asian studies.

Another important difference is that, while upfront surgery – to which adjuvant chemotherapy is added – is the standard treatment of gastric cancer in the East, a multimodal approach with addition of perioperative chemotherapy has been established as the current state-of-the-art treatment in the West [4, 26]. Unfortunately, very few studies report the number of patients which received neoadjuvant treatment. In a small-scale RCT analyzing the impact of neoadjuvant chemotherapy for AGC in the surgical outcome, Li et al. showed that the group of patients treated laparoscopically did benefit of the lower risk of postoperative complications and were able to better tolerate the planned adjuvant chemotherapy [27]. Nevertheless, evidence on the role of minimally invasive surgery in patients treated with neoadjuvant chemotherapy is still lacking and further investigation comparing laparoscopic and open gastrectomy in the era of perioperative therapies is warranted. Two European multicenter RCTs, the LOGICA- trial and the STOMACH trial, have been launched and their results are anticipated [28, 29].

Our department started applying laparoscopic surgery for the treatment of gastric cancer in 2012, and during the years that followed we gradually extended our indications from EGC in the distal part of the stomach, to AGC and finally to tumors mandating a total gastrectomy or even multivisceral resection in selected cases. During this latter study period, extended bursectomy has been abandoned [30] and splenectomy performed much more selectively [31], allowing us to expand the indications for laparoscopic resection. These alterations significantly affected the learning curve in many centers, including our own. As a result, in the period 2016–2019 the majority of patients who were operated laparoscopically had been diagnosed with AGC, and LTG with more extended en bloc lymphadenectomy was frequently performed. The latter was expressed in terms of higher intraoperative blood loss and longer operation time and explains at the same time the higher number of harvested lymph nodes (Fig. 4). Furthermore, surgical training of two younger colleagues in the department may be another factor affecting these findings.

**Fig. 7:**
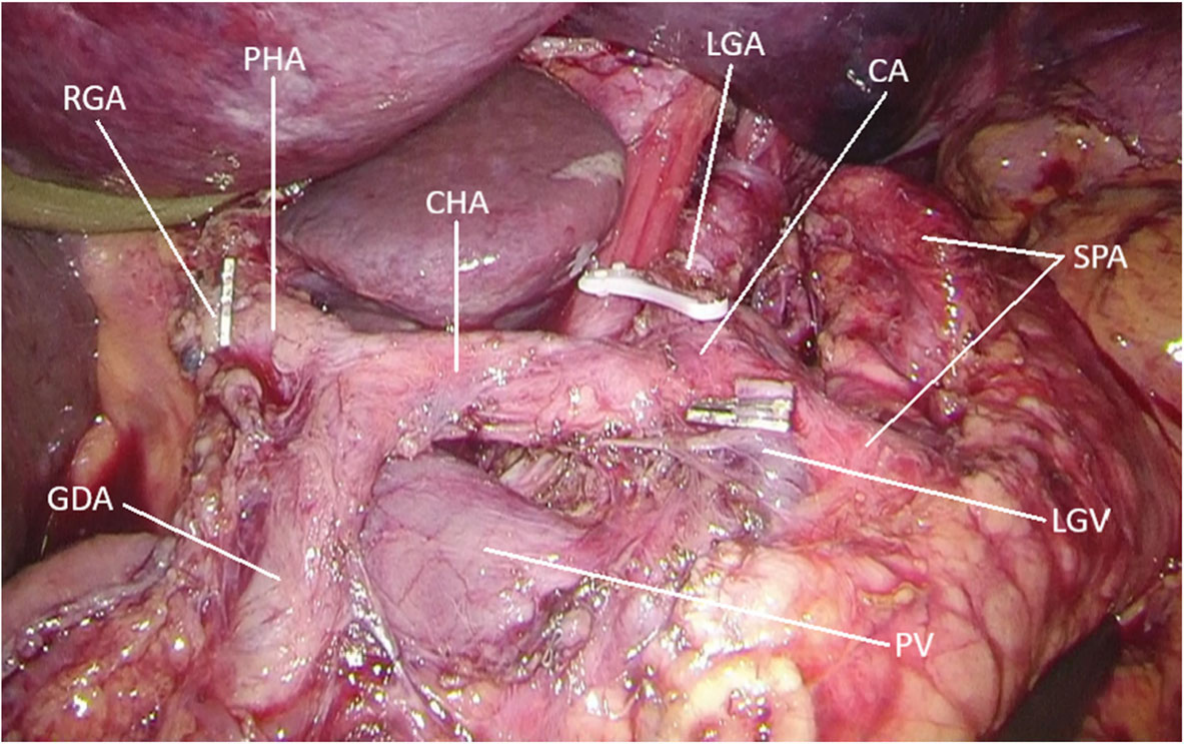
Intraoperative capture after completed lymph node dissection along the branches of the celiac trunk. The left and right gastric arteries are divided at their origin

Our results during the implementation period were recently reported in detail [32]. Despite the retrospective design of the study, the two patient groups were well balanced with regard to baseline characteristics (age, gender, mean BMI, ASA score, clinical T- and N-stage and the proportion of patients receiving neoadjuvant chemotherapy). The analysis showed fewer severe complications (Clavien-Dindo ≥IIIb) with lower anastomotic leak rate, shorter hospital stay and no 30- and 90-day mortality in the laparoscopic group. Supplementary analysis regarding the impact of patients’ characteristics (such as age, gender, BMI and neoadjuvant chemotherapy) did not indicate any additional difficulty in implementing the technique in a specific subgroup of patients. Furthermore, no difference in overall survival was found. We have also been able to confirm that the oncological requirements comprising adequate lymphadenectomy and negative resection margins can be met (Fig. 7), given that surgery is performed in centers with adequate experience. Indeed, in the majority of trials from Asia (i.e. KLASS-01, KLASS-02, JCOG0912, JLSSG0901, CLASS-01), strict requirements were applied regarding the case volume of institutions and the experience of participating surgeons. Especially in Japan, in order to guarantee surgical quality, it was required that the participating surgeons were certified by the Japan Society for Endoscopic Surgery (JSES) according to the Endoscopic Surgical Skill Qualification System [33]. This emphasizes the importance of a high case volume in order to develop and maintain the necessary surgical skills and perioperative routines for such demanding procedures; in those terms, and in particular in a low incidence country as Sweden, the contribution of the centralization of UGI-cancer surgery has been essential.

Due to our limited case volume, as in most Western centres, and in order to accelerate the learning process, at least two of the three senior consultants participated in the first 50 laparoscopic operations. Usually, one consultant was performing the resection and the other the reconstruction part of the procedure. Thus, we are unable to provide information with regards to individual surgeon’s learning curve and the results in the current article reflect the experience of the whole team as such.

Acquiring the Japanese surgical principles and expertise has been an ongoing and evolving initiative and has resulted in the successful implementation of laparoscopic gastric cancer surgery at our institution. Apart from this collaboration, centralization of gastric cancer surgery that has occurred in Sweden almost simultaneously, has resulted in an increased case load which in turn provided the conditions for better quality research and establishment of fast-track pathways.

## Conclusions

Implementation of minimally invasive surgery for gastric cancer at a tertiary referral center was a process that required thoughtful steps over time in order to gradually and safely establish the technique. Exchange of surgical experience and knowledge in collaboration with two high-volume centers in Japan has been of paramount importance on a long pathway that has strictly followed the oncological principles as the main endpoint of this adaptive process. This effort has led to a successful incorporation of laparoscopic surgery, which has become the procedure of choice for patients offered a curative treatment.

## Data Availability

Availability of data and materials: This is a retrospective review of prospectively collected data retrieved from the Karolinska University Hospital’s surgical planning system (ORBIT) and cross-matched for validation with the patient chart system (Take Care). The datasets used and analyzed during the current study are available from the corresponding author upon reasonable request.

## Abbreviations

EGC: Early gastric cancer
AGC: Advanced gastric cancer
LDG: Laparoscopic distal gastrectomy
UGI-surgery: Upper gastrointestinal surgery
AMC: Academic Medical Center
LTG: Laparoscopic total gastrectomy
DFS: Disease-free survival
JSES: Japan Society for Endoscopic Surgery
